# Removing the Esophageal Stump During Reconstruction for Esophagojejunostomy in Total Gastrectomy for Gastric Cancer: the Modified Overlap Method

**DOI:** 10.1007/s11605-023-05600-4

**Published:** 2023-01-30

**Authors:** Yoshihiko Kakiuchi, Shinji Kuroda, Satoru Kikuchi, Hajime Kashima, Masahiko Nishizaki, Shunsuke Kagawa, Toshiyoshi Fujiwara

**Affiliations:** 1 Department of Gastroenterological Surgery, Dentistry and Pharmaceutical Sciences, Okayama University Graduate School of Medicine, 2-5-1 Shikata-Cho, Kita-Ku, Okayama, 700-8558 Japan; 2 Department of Surgery, Tsuyama Chuo Hospital, 1756 Kawasaki, Tsuyama City, Okayama, 708-0841 Japan

## Introduction

The first laparoscopic gastrectomy for gastric cancer was performed in 1991, as a laparoscopic-assisted surgery with reconstruction performed through a midline epigastric incision. ^1^ Since that report, minimally invasive surgical approaches have been the focus of primary approaches for gastric cancer treatment as alternatives to the traditional open approach. Although the JCOG1401 study by the Japan Clinical Oncology Group ^2^ showed the safety of laparoscopic-assisted total gastrectomy (TG), including in terms of anastomotic-related complications, we consider the technical difficulty of esophagojejunostomy as an issue in need of further improvement.

Based on the concept of body tissue protection, we developed a new anastomosis using the linear stapler (LS) technique: the modified overlap method. Removal of the staples of the esophageal stump differs in this method compared to the conventional overlap method. The esophageal stump is often grasped by the forceps to achieve a clear surgical field, but this can result in injury to the esophageal stump without the surgeon noticing. Our method may decrease the risk of complications caused in such events. Herein, we describe the technique and therapeutic outcomes.

## Materials and Methods

### Patients

Between June 2016 and December 2021, totally laparoscopic TG (LTG) or robot-assisted TG (RTG) with our method was performed for 66 patients with gastric cancer at Okayama University Hospital in Japan (Table 1). We retrospectively reviewed and evaluated patient records for postoperative complications.

**Table 1:** Clinicopathological characteristics

*N*	66
Age, years	
Median (IQR)	69 (63–75)
Sex	
Male	47
Female	19
Approach	
Laparoscopy	52
Robot	14
Operative time, min	
Median (IQR)	420 (351–496)
Blood loss, mL	
Median (IQR)	100 (50–298)
Postoperative hospital stay, days	
Median (IQR)	12 (11–15)
Lymph node dissection	
1	3
1 +	14
≥ 2	49
Pathological clinical stage	
I	16
II	23
III	23
IV	3
Uncategorized	1

### Surgical Technique

**Fig. 1:**
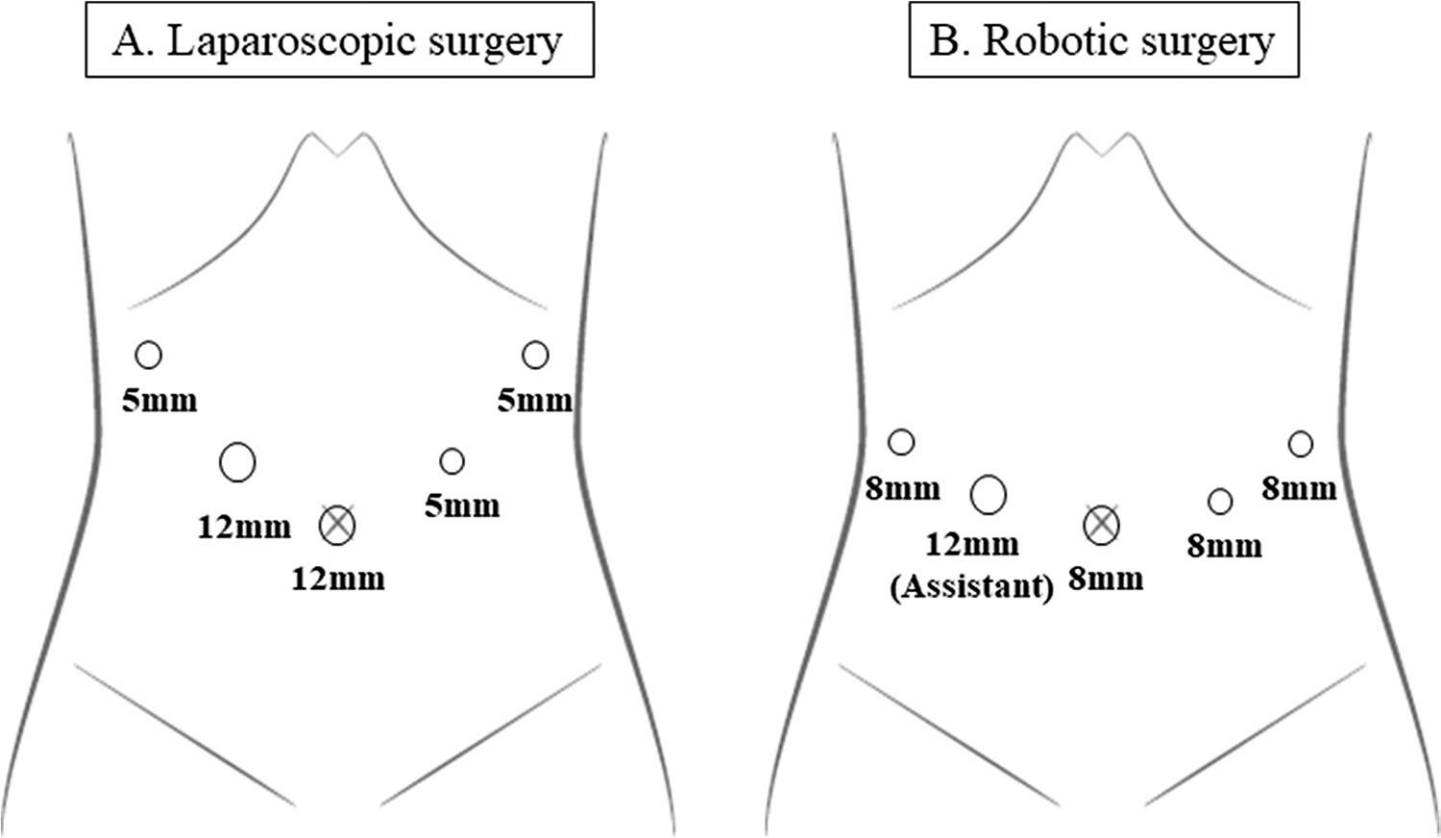
Positions of the surgical ports in the abdomen. **A** Positions in laparoscopic surgery. **B** Positions in robotic surgery

**Fig. 2:**
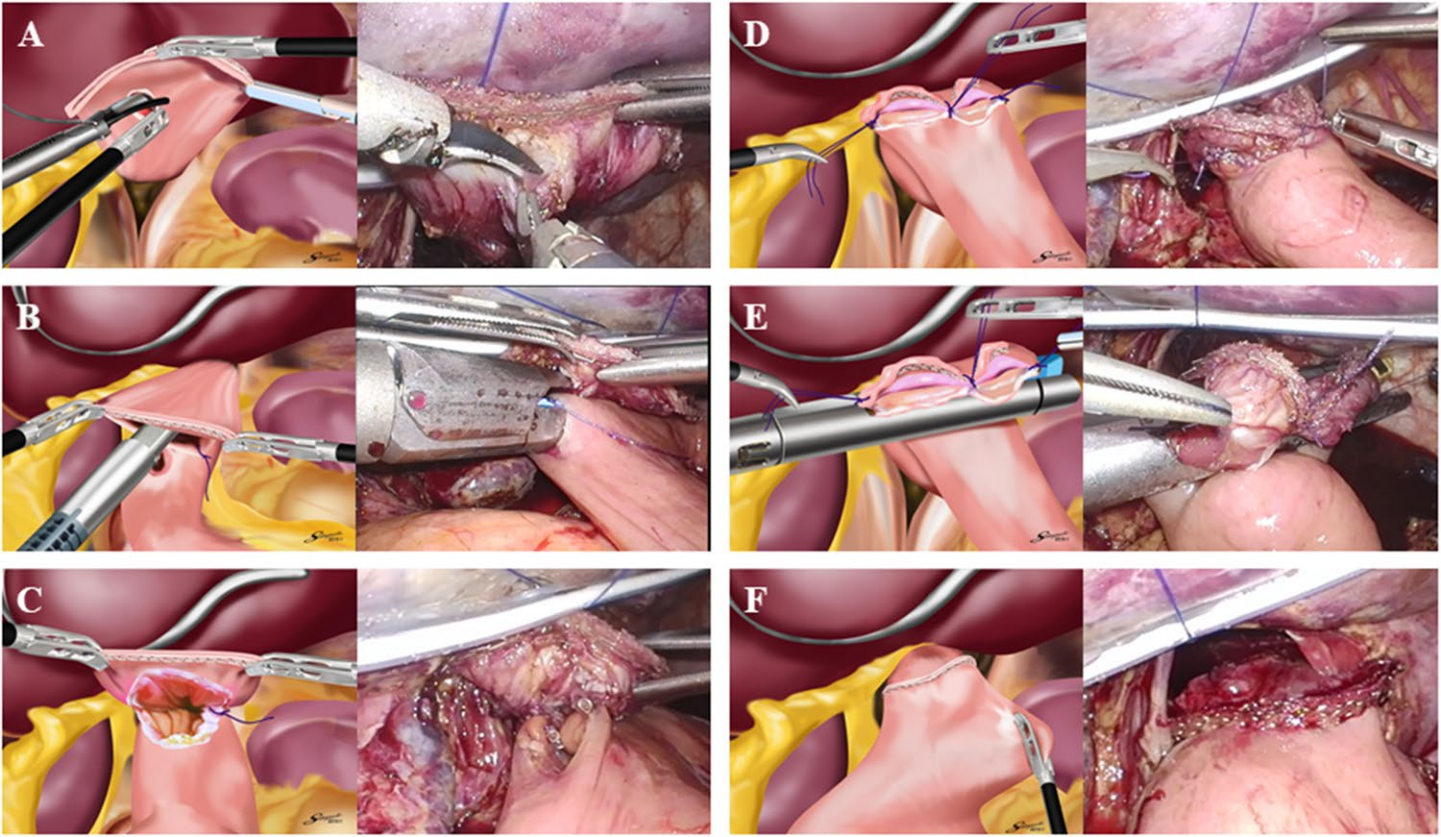
Reconstruction procedure for the modified overlap method. **A** The surgeon makes a hole at the center of the esophageal posterior wall. **B** After creating a small entry hole in the jejunum, we confirm the insertion of the linear staples and suture. **C** Hemostasis inside the anastomosis is confirmed. **D** Both edges and the center of the common entry hole are sutured. **E** The common entry hole and both edges of the esophageal stump are sandwiched using the LS. **F** Finished shape

For LTG, a laparoscope was inserted via a 12-mm median umbilical port, and 4 operating ports were placed on both sides of the upper abdomen (Fig. 1A). After lymph node dissection and transection of the duodenum, soft tissue and vagus nerves around the esophagus were removed. The surgeon transected the esophagus with an ENDOPATH® STAPLER Powered ECHELON FLEX® (Ethicon Endo-Surgery Inc., Cincinnati, OH, USA) equipped with GST® System blue 60 mm (Ethicon Endo-Surgery Inc.). At this time, we recommended using a buttress material such as ECHELON ENDOPATH® Staple Line Reinforcement (SLR; Ethicon Endo-Surgery Inc.), and the specimen was extracted via a small incision at the umbilicus. The assistant kept holding both edges of the esophageal stump, and the surgeon made a hole in the center of the esophageal posterior wall (Fig. 2A). At this point in the procedure, we recommended that the assistant grasp the SLR to avoid tissue injury. The mucosa and muscular layer of the esophageal posterior wall were sutured with 3–0 VICRYL PLUS® (Ethicon Endo-Surgery Inc.) at a single point to prevent the mucous membrane from slipping. A small entry hole was made on the antimesenteric side. A stapler cartridge was inserted into the jejunal entry hole, and the anvil was inserted into the esophageal entry hole. After these insertions, we performed suturing using the LS (Fig. 2B). After confirming hemostasis on the inside of the anastomosis (Fig. 2C), both edges and the center of the common entry hole were sutured with 3–0 VICRYL PLUS (Fig. 2D). When we sutured the jejunum and esophagus, the esophageal side was sutured to the esophageal anterior wall beyond the stapled esophageal stump. While lifting up the three threads of the sutures, the common entry hole and both edges of the stapled esophageal stump were sandwiched using a 60-mm Powered Echelon Flex (Fig. 2E) and closed (Fig. 2F).

For RTG, a scope was inserted into the abdominal cavity through an 8-mm port at the umbilicus. Two more operating ports (8 mm) were placed at bilateral hypochondriac regions and a further port (8 mm) was placed on the left lateral side. Finally, an assistant port (12 mm) was located on the right lateral side (Fig. 1B). The reconstruction procedure was almost the same as in LTG, whereas the assistant surgeon carried out procedures such as resection and suturing using the LS.

## Results

Only one case of anastomotic leakage (1.5%) was encountered as a postoperative anastomosis-related complication, along with two cases of pancreatic fistula (3.0%) and one case of intraperitoneal hemorrhage (1.5%) as non-anastomosis-related complications (Table 2).

**Table 2:** Postoperative complications

*N*	66
Anastomosis-related complications (%)	
Leakage	1 (1.5%)
Stricture	0
Bleeding	0
Non-anastomosis-related complications ≥ C-D Grade III	
Pancreatic fistula	2 (3.0%)
Intraabdominal infection	0
Intraperitoneal hemorrhage	1 (1.5%)

## Discussion

The overlap method generally preserves the esophageal stump, including staples. However, our method differs in that the stump is cut off. One advantage of our method is the ability to grasp the esophageal stump with the staple. This advantage can lead to flexible deployment of the surgical field without needing to worry about esophageal injury. As a result, we can provide a clear surgical field when the anastomosis is performed, particularly the left side of the anastomosis, which tends to be obscured. Another advantage is that this method does not require advanced suturing skills because the entry hole is closed by the LS. Closure by the LS in addition to the total of 3 solo sutures, rather than the continuous sutures, leads to a reduced risk of anastomosis leakage. This method prevents unintentional suturing of the posterior wall because the anterior walls of the esophagus and jejunum are only sutured after being lifted and thus clearly separated from the posterior wall. Furthermore, while continuous sutures may cause stenosis by unintentionally stitching the posterior wall or by tightening the thread too tightly, this method reduces such risks.

Our method of removing the esophageal stump with the staples can facilitate reconstruction and represents a safe, feasible method for reconstruction following TG. We believe that our technique will contribute to the spread of safer LTG and RTG with fewer complications.

## Supplementary Information

Below is the link to the electronic supplementary material.

Supplementary file1 (MP4 287998 KB)

## Data Availability

The datasets used during the current study are available from the corresponding author on reasonable request.
